# Perturbation on gut microbiota impedes the onset of obesity in high fat diet-induced mice

**DOI:** 10.3389/fendo.2022.795371

**Published:** 2022-08-09

**Authors:** Zhongjia Yu, Xiang-Fang Yu, Goher Kerem, Pei-Gen Ren

**Affiliations:** ^1^ Center for Energy Metabolism and Reproduction, Shenzhen Institute of Advanced Technology, Chinese Academy of Sciences, Shenzhen, China; ^2^ Shenzhen College of Advanced Technology, University of Chinese Academy of Sciences, Shenzhen, China; ^3^ Xinjiang Key Laboratory of Special Species Conservation and Regulatory Biology, College of Life Science, Xinjiang Normal University, Urumqi, China

**Keywords:** jejunal microbiota, obesity, T2DM, perturbation, bioinformatic pipelines

## Abstract

High-calorie intake has become one of the most common causes of dietary obesity, which eventually develops into type 2 diabetes mellitus (T2DM). Microbiota, along with the length of the gastrointestinal tract, is related to metabolic disorders, but its shifts and following impact on metabolic disorders due to external perturbation are still unclear. To evaluate shifts of microbiota from the proximal to the distal intestine and their impact on metabolic disorders, we profiled jejunal and colonic microbiota with the perturbation using high salt (HS) and antibiotic-induced microbiota depletion (AIMD) in diet-induced obesity (DIO) mice and analyzed the association with parameters of both obesity and blood glucose. After ten weeks of feeding DIO mice with HS intake and AIMD, they failed to develop obesity. The DIO mice with HS intake had T2DM symptoms, whereas the AIMD DIO mice showed no significant difference in blood glucose parameters. We observed that the jejunal and colonic microbiota had shifted due to settled perturbation, and jejunal microbiota within a group were more dispersed than colonic microbiota. After further analyzing jejunal microbiota using quantified amplicon sequencing, we found that the absolute abundance of *Colidextribacter* (R = 0.695, p = 0.001) and *Faecalibaculum* (R = 0.631, p = 0.005) in the jejunum was positively correlated with the changes in BW and FBG levels. The predicted pathway of glucose and metabolism of other substances significantly changed between groups (p <0.05). We demonstrated that the onset of obesity and T2DM in DIO mice is impeded when the gut microbiota is perturbed; thus, this pathogenesis depends on the gut microbiota.

## Introduction

Dietary obesity has raised worldwide public health concerns (1), for which diet-induced obesity (DIO) mice are the most commonly used animal model. The etiology of HFD-induced obesity has been found to be associated with gut microbiota. In previous studies, germ-free mice fed with HFD failed to manifest obesity but became obese only after fecal microbiota transplantation (FMT), indicating that gut microbiota play an irreplaceable role in dietary obesity (2, 3). Shifted by diets, gut microbiota, in turn, can influence the progress of obesity forming. For instance, HFD favored bacteria fermenting end products, such as SCFAs, providing extra energy for body fat depositing (4). Moreover, gut microbiota affect metabolism by modulating gut peptide signaling and causing low-grade inflammation (4). Most studies have extended the impacts of microbiota on the metabolism in HFD-induced obesity through microbiota depletion *via* germ-free animals and antibiotics inducing (2, 5). In this way, the existence of gut microbiota for HFD-induced obesity has been proven to be necessary. However, since the gut microbiota signatures are susceptible to many factors, including diet (6–8), gut microbiota profiles may vary depending on perturbation, and they may affect the development of obesity and related disorders, for instance, T2DM. In the southwestern region of China, diets high in salt are very typical. Nevertheless, neither the profiles nor their correlation to obesity of gut microbiota perturbed by high salt intake have been well studied.

Gut microbiota is the sum of bacteria in the intestinal tract, from the duodenum to the rectum. Fecal microbiota is primarily studied in medical research, often referred to as gut microbiota, since feces samples are easy to obtain and are the source for FMT (9). In mice models, cecal, colonic content, and feces are mainly used to study gut microbiota as these materials have more similar gut microbiota and they are good mocks (10, 11). Therefore, content in the large intestines and feces became the first choices. In HFD-induced obesity, microbiota in feces and large intestines showed that deep digestion of fibers is the primary cause of the bacterial contribution to obesity (4). However, the large intestine only absorbs the remaining substrates that are not absorbed in the small intestine. Thus, the energy nutrients produced by bacteria in the large intestine may be overestimated to upset the energy balance of the body. In contrast, the small intestine is in charge of food digestion and absorption, especially the jejunum, but microbiota in these sites are rarely focused on because sampling is a challenge in clinical practice. For this reason, although microorganisms in the small intestine have been proven to play crucial roles in transducing dietary signals and inducing the alterations of the bacterial population and intestinal abnormalities, the role of microbiota in the small intestine is still underestimated (12). Small intestine microbiota was demonstrated as a regulator for dietary lipids digestion and absorption (13). However, diets could shape microbiota in the whole digestive tract, of which the size of the effect remains unclear for each part.

Analyzing gut microbiota based on next-generation sequencing has become a standard workflow. 16S rRNA amplicon sequencing can display the microbiota as much as possible economically, although the identification of precise bacteria is limited due to the partial amplified regions. The outcomes obtained for amplicon sequencing are primarily dependent on multiple factors, such as DNA extraction, library construction, and bioinformatics analysis pipelines. The influence of some factors on final results has been assessed in many studies (14–16), but few studies have compared the outcomes of their sequencing data analyzed using different bioinformatic pipelines, which are the most personal choice. Moreover, the advantage of absolute abundance has recently been noted to assess the changes of a specific taxon, and the calculation based on copies quantified by qPCR is the easiest to perform (17). However, a minimal study has applied absolute abundance to reveal the differences in gut microbiota in HFD-induced obesity.

We evaluated the shifts of gut microbiota in both the small and large intestines of DIO mice using perturbation with antibiotic-induced microbiota depletion (AIMD) and high salt intake, respectively, and the impact of the above shifts on the onset of obesity and T2DM in these mouse models. Besides, we quantificationally analyzed jejunal microbiota to specify their signatures related to HFD-induced obesity and illustrated that the function of primary nutrition metabolism occurs in the proximal small intestine. Commonly used bioinformatics pipelines and parameters were co-conducted for more objective interpretation in this study.

## Materials and methods

### Animals and study design

**Figure 1:**
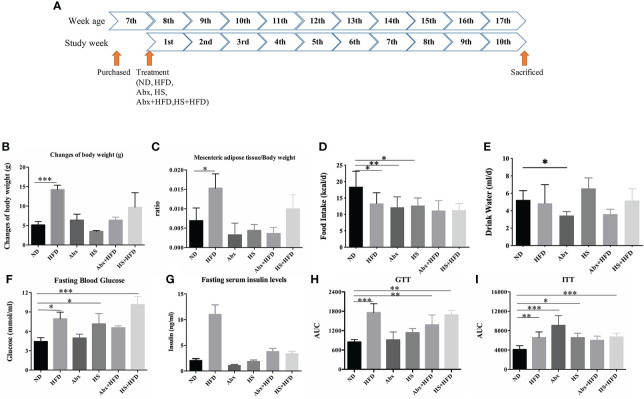
Measures of obesity and T2DM (n = 5). **(A)** schema of study design; **(B)** changes in B.W.; **(C)** ratio of mesenteric adipose tissue to B.W.; **(D)** food intake; **(E)** Water intake; **(F)** fasting blood glucose level; **(G)** fasting serum insulin level; **(H)** area under curve of glucose tolerance test; **(I)** area under curve of insulin tolerance test. * p<0.05, ** p<0.01, *** p<0.001.

Seven-week-old male SPF C57BL/6N mice (Charles River Laboratories, Beijing) were purchased and housed in an SPF environment with free access to food and water. After a one-week adaptation, the mice were randomly divided into six groups (each group: n = 5), including a normal diet (ND) group, a high-fat diet (HFD) group, antibiotic-induced microbiota depletion (Abx) in the ND group, high salt intake (HS) in the ND group, Abx with HFD (Abx + HFD) group, and HS with HFD (HS + HFD) group. For the normal diet, 65.08% of energy is from carbohydrates, 23.07% from protein, and 11.85% from fat. For HFD, feed containing 45% energy from fat (D12451; Research Diets, New Brunswick, New Jersey) was used. For AIMD, drinking water was mixed with ampicillin (1 mg/ml; A102048, Aladdin, China), vancomycin (0.5 mg/ml; V105495, Aladdin, China), neomycin (0.5 mg/ml; N109017, Aladdin, China), metronidazole (1 mg/ml; M109874, Aladdin, China), and amphotericin B (0.5 μg/ml; A105482, Aladdin, China) during the experiments (18). For HS intervention, drinking water was added with 2% NaCl during the experiments. Filtered sterile drinking water was supplemented with 0.01% aspartame (Nantong Changhai Food Additive Co. Ltd., China) to produce better palatability. During the experiment, body weight (BW), food intake, and water intake were recorded weekly. All mice in each group were sacrificed until fed for ten weeks, as shown in **Figure 1A**. The weight of the mesenteric adipose tissue of each mouse was measured. All experimental animal-related procedures were approved by the Ethics Committee for Animal Research, Shenzhen Institutes of Advanced Technology, Chinese Academy of Sciences (protocol number: SIAT-IRB-170401-YGS-RPG-A0312-01).

### Fasting blood glucose, glucose tolerance test, and insulin tolerance test

The murine obesity-T2DM model was evaluated using FBG, GTT, and ITT. Mouse tail snip blood samples were used for FBG measurement (blood glucose strips and Accu-Check glucometer, Roche, Switzerland). The animals were fasted for 5 h with free water before the FBG test. For GTT, the mice were pre-fasted for 16 h and then injected with glucose solution (2 g/kg·BW) intraperitoneally (i.p.). Blood glucose was then measured at different time points as 0, 15, 30, 60, 90, and 120 min, respectively. ITT was performed after 4-hour fasting, with insulin (0.75 U/kg, Humulin, Lilly, USA) i.p. injected, and then blood glucose was measured at the indicated time points as in GTT.

### Serum insulin level

After 10 weeks of feeding, the mice were anesthetized by isoflurane (RWD Life Science, China) before euthanization. Blood samples were collected by the enucleation technique from mice under anesthesia, maintained at room temperature for 2 h, and centrifuged (1,500 rpm at 4°C) for 30 min to separate serum. Serum insulin concentrations were determined by the Rat/Mouse Insulin 96 Well Plate Assay (MERCK, Germany).

### Sample preparation and 16S rRNA amplicon sequencing

The colonic content and jejunum (each group: n = 5) were collected sterilely according to a previous study (13). Briefly, 1 cm of the jejunum with its content was put into individual sterile Eppendorf after discarding 2 cm of the jejunum next to the duodenum. Then, the tissues were snap-frozen in liquid nitrogen and then stored at −80°C until experiments.

All samples were shipped to Tinygene Company (Shanghai, China) for the 16S rRNA amplicon sequencing. Briefly, genomic DNA was extracted using the QIAamp DNA Stool Mini Kit (QIAamp, Germany) according to the instructions of the manufacturer. Extracted DNA was purified using the AxyPrepDNA kit (Axygen, Corning, China). Primers 341F (CCTAYGGGRBGCASCAG) and 806R (GGACTACNNGGGTATCTAAT) were used to amplify the V3–V4 region of the bacterial 16S rRNA gene, and then each target fragment was recovered as the template for a secondary round of PCR amplification. The samples were mixed according to the mole ratio to complete library preparation before sequencing. For amplicon sequencing, DNA libraries of pair-ends with a single index were constructed using the Truseq-DNA-PCR-free-library-prep kit. Sequencing was performed on an Illumina Miseq PE 300 platform. For jejunal samples (each group: n = 3), PCR products were recovered (AxyPrep DNA gel recovery kit, Axygen, USA) and quantified with an FTC-3000TM real-time PCR instrument (Funglyn Maple Ridge, Canada).

### Bioinformatics analyses

The four commonly used 16S rRNA amplicon sequencing analysis pipelines (DADA2, Deblur, Usearch_UNOISE3, and Usearch_UPARSE) were used to generate Amplicon Sequence Avariants (ASVs) or Operational Taxonomic Units (OTUs) for the following analyses, and the schema is shown in **Supplementary Figure 1**.

DADA2: Raw data were analyzed using the QIIME2 (version 2021.02) software pipeline. Reads were demultiplexed with q2-demux. Then, the DADA2 plugin was implemented for the quality control process, and all phiX reads and chimeric sequences were filtered. Based on the demux summary, sequences of forwarding were truncated to a length of 270 bases and reverse reads to a length of 250 bases. After denoising, the data were obtained using the DADA2 denoise-paired method, representative sequences of each sample were retained.

Deblur: raw data were also analyzed in the QIIME2 pipeline. Pair end reads were joined using search after the raw reads were demultiplexed with q2-demux. Then, the deblur plugin was implemented for quality control processing, and all phiX reads and chimeric sequences were filtered. Reads with lengths longer than 170 bp were retained for representative sequence generation.

Usearch_UNOISE3: raw data were processed in the Research 11 pipeline. Reads were merged using fastqout, followed by primer removal and quality checking. A minimum length of 400 bp and a maximal length of 480 bp were retained for the following process. The UNOISE3 method was used for generating ASVs.

Usearch_UPARSE: reads were processed until denoising as per the UNOISE3 pipeline. Then, the Cluster_OTU method was used to generate OTUs.

After raw data were processed, all ASV (OTU) files and tables, either from QIIME2 or from research pipelines, were imported into QIIME2 for the following processing. ASVs (OTUs) out of four pipelines were filtered out of non-bacterial sequences against Silva 99% and Greengene 99% databases on 97% identity using method search, respectively. Then, Naive Bayes classifiers pre-trained on Greengenes 13_8 99% OTU full-length sequences and Silva 138 99% OTU full-length sequences were used correspondingly for taxonomy assignment. Afterward, due to the fact that the copies of 16S rRNA are not distributed equally among different bacteria, the normalization of copies was conducted based on the taxonomic information using the settled plugin in QIIME2.

### Statistical analyses

Energy intake was calculated according to the net amount of food intake and the physiological fuel values of the ingredients. The data of BW and FBG were normalized based on energy intake. All enumeration data were checked for normality and homogeneity of variance using the Shapiro–Wilk test and Levene’s test. A one-way ANOVA test was used to assess the significance of the difference. SPSS 20.0.0 was applied to these statistical analyses. Reported significant differences are corrected for multiple testing using the Benjamini–Hochberg correction with a 5% false discovery rate. The chi-square test was applied for the correlation assessment.

The significance of the difference in bacterial richness between groups was assessed using microeco (19). ImageGP was applied to the analysis of alpha diversity and principal coordinate analysis (PCoA) (20). Differences in the abundance of each taxon between groups were analyzed using DESeq (21). Metabolic pathways were predicted using PICRUSt, and the significance of differences was analyzed using one-way ANOVA in STAMP (22, 23). All plots were generated using ggplot2 (24) through R 3.6.1 (25).

## Results

### Obesity and T2DM symptoms

We normalized the data of BW changes and FBG based on the entire energy intake to reduce the impact of the amount of food intake. As shown in **Figures 1B, C**, only HFD mice showed a significant increase in body weight (p = 0.00001) and a significantly higher ratio of mesenteric adipose tissue to BW (p = 0.03) after 10 weeks, and no significant difference was observed in food and water intake between DIO groups (**Figures 1D, E**). The mice treated by either HS or antibiotics, regardless of HFD, did not display any significant changes in BW or a higher ratio of adipose tissues to BW, suggesting a perturbation of gut microbiota limited HFD-induced obesity.

HFD mice successfully developed T2DM symptoms, as the mean FBG levels were at 8.01 mmol/ml (p = 0.01) with a significantly higher serum insulin level (p = 0.000037) and a higher area under the curve (AUC) of GTT (p = 0.000001) and ITT (p = 0.004) than the ND group (**Figures 1F–I**). Although the same group was fed with HFD, the Abx group (Abx + HFD) only had a significantly higher AUC of GTT (p = 0.004) than the ND group, with the highest FBG level of 6.55 mmol/ml, suggesting this group failed to develop complete T2DM. The HS + HFD group succeeded in developing T2DM, including a mean FBG level of 10.14 mmol/ml, significantly higher glucose tolerance (p = 0.000032), and insulin tolerance (p = 0.011). Moreover, single perturbation with either antibiotic (p = 0.00002) or HS (p = 0.013) significantly increased the insulin tolerance of the mice than ND (**Figure 1I**).

### Comparison of pipelines for bioinformatics analyses

We assessed four commonly used pipelines using our quantified 16S amplicon sequencing data from the jejunum (n = 3 for each group). The general information is listed in **Supplementary Table 1**.** **A total of 748,154 raw reads were obtained from amplicon sequencing, followed by 45.43% of reads passing denoising with DADA2, 34.51% with Deblur, 50.13% with UNOISE3, and 49.76% with UPARSE. No significant difference in the number of reads was observed between DADA2, UNOISE3, and UPARSE, while the number of reads was significantly decreased after denoising with Deblur (versus DADA2: p = 0.002; UNOISE3: p = 0.000015; UPARSE: p = 0.000023). ASVs/OTUs were generated with the above four denoisers and then filtered out non-bacterial or unknown reads against the Silva and Greengenes databases, respectively, as shown in **Table 1**. The highest ASVs/OTUs number was generated with Deblur (n = 856), followed by DADA2 (n = 535), UPARSE (n = 243), and UNOISE3 (n = 89). By comparing the two taxonomy databases, Silva identified a few more bacterial sequences than Greengenes concerning the number of ASVs/OTUs and hit reads. More reads that failed to hit bacterial sequences were observed in DADA2; in other words, more unknown unique sequence variants were detected using this denoiser.

**Table 1:** Counts of reads and ASVs (OTUs) after denoising and filtering.

	Denoised Reads	ASVs (OTUs)	Silva				Greengene			
			ASVs (OTUs)	Taxon	Hits reads	Missed reads	ASVs (OTUs)	Taxon	Hits reads	Missed reads
DADA2	339,931	535	426	144	293,856	46,075	424	111	293,282	46,649
Deblur	258,152	856	777	62	248,687	9,465	761	67	245,564	12,588
UNOISE3	375,058	89	86	44	372,747	2,311	85	36	372,216	2,842
SPARSE	372,318	243	232	163	371,888	430	230	125	371,324	994

In-depth analyses of diversity measures using outcomes from these four pipelines are shown in **Supplementary Figure 2**. As UNOISE3 with Silva provided us with fewer ASVs and more common diversity features among these pipelines, we analyzed our data completely using this pipeline. Data normalized for 16S rRNA copies were used for relevant statistical analyses.

### Comparison of shifts in jejunal and colonic microbiota

**Figure 2:**
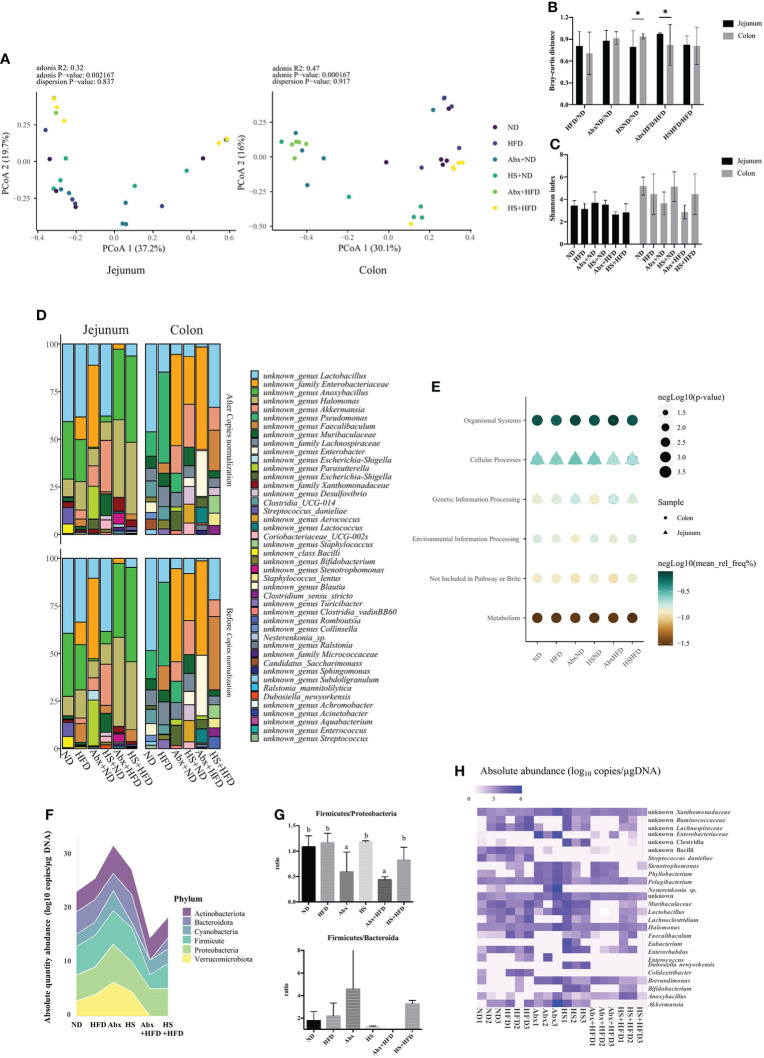
Diversity metrics and predicted metabolic pathway of gut microbiota. **(A)** PCoA analysis for jejunal and colonic microbiota; **(B)** Bray–Curtis distance between groups; **(C)** Shannon index. **(D)** Top 10 taxa identified. **(E)** Significantly different pathway between groups at KEGG Level-1; **(F)** the absolute abundance of the phylum between group; **(G)** ratio of Firmicutes to Proteobacteria and Firmicutes to Bacteroidetes; **(H)** Significantly different bacteria between groups. **(A–E)** n = 5 for each group; **(F–H)** n = 3 for each group, only jejunal microbiota. *p<0.05

Although both jejunal and colonic microbiota profiles showed significant differences between groups (adonis p <0.01), jejunal microbiota was more dispersed within a group than the colonic microbiota (**Figure 2A**), which suggests that the shifts of jejunal microbiota facing the same perturbation were uncertain. By comparing the effect size of perturbation on both microbiota profiles, we observed that high salt with ND and antibiotics with HFD had a significantly different impact on jejunal and colonic microbiota (**Figure 2B**). In other words, antibiotics affected more colonic microbiota than the jejunum in DIO mice, while high salt affected the jejunum. A high-fat diet reduced gut microbiota richness in both the small and large intestines and increased antibiotics and high salt content (**Figure 2C**).

The top 10 bacteria in each group and each site are present in **Figure 2D**. Generally, the genus Lactobacillus was one of the top 10 abundant bacteria, whose relative abundance changed dependent on the perturbation, and the same trend could be observed for the family Enterobacteriaceae in both the jejunum and colon. The 16S copy normalization had a minimal impact on the most abundant bacteria. We predicted the function of microbiota at both sites using PICRUSt and found that the metabolism of colonic microbiota significantly changed dependent on perturbation, whereas other significantly changed pathways in both jejunal and colonic microbiota were related to host interaction, especially cellular processes that were tightly linked with the jejunal microbiota (**Figure 2E**).

### High salt intake and AIMD changed the diversity of jejunal microbiota

Since the jejunum is the main site for nutrition absorption and its microbiota had higher uncertainty facing perturbation, we in-depth analyzed jejunal microbiota using quantified 16S amplicon sequencing. As shown in **Supplementary Figure 2A**, the richness of jejunal microbiota decreased in both the HFD and perturbation groups, while the AIMD mice fed with HFD had significantly lower richness (p <0.05). However, the Shannon index between groups had no significant difference, although it was higher in DIO and AIMD mice compared to the ND group. ANOSIM revealed that the dissimilarity of bacterial communities between groups was significantly different from the dissimilarity of communities within a group (R = 0.49, p = 0.001), suggesting that each group had a unique jejunal microbiota signature. Compared to ND, both diet and perturbation of HS and antibiotics shifted the jejunal microbiota of mice. Distinct jejunal microbiota signatures between groups could be observed in the PCoA plot (**Supplementary Figure 2B**). Abx + HFD, HS, and HS + HFD groups had similar jejunal microbiota signatures within a group, whereas ND, HFD, and Abx groups had distinct ones. Moreover, antibiotics conspicuously reshaped the bacterial composition, as the most dominant bacteria in the jejunum shifted to the family *Enterobacteriaceae* from *Lactobacillus*, and the latter was the most dominant in other groups (**Supplementary Figure 2C**).

As shown in **Table 2**, we summarized the taxa with a significant difference between normal diet and other groups, and the limitation of the relative abundance to find different bacteria compared to the absolute abundance was noticed. Thus, we used the dataset with absolute abundance in the following analyses. Total bacterial copies in per micro-gram DNA were higher in Abx followed by HS and HFD but decreased in Abx + HFD and HS + HFD group (**Figure 2F**) compared to ND, indicating the combination of high-fat diet and perturbation for microbiota reduced the bacterial amount. Moreover, antibiotics significantly changed the ratio between Firmicutes and Proteobacteria regardless of diets (**Figure 2G**). However, the ratio between Firmicutes and Bacteroides had no significant difference between these groups, except for the Abx + HFD group, in which there were no Bacteroides identified (**Figure 2G**). The absolute abundance of 25 taxa was significantly different between ND and other groups, as shown in **Figure 2H**. A significantly higher absolute abundance of *Bifidobacterium* (p = 3.45 × 10^−12^), *Faecalibaculum* (p = 0.00005), and *Stenotrophonomas* (p = 0.006) was found in HFD mice, while a significantly lower absolute abundance of *Parasutterella* (p = 0.0000133) and unknown *Enterobacteriaceae* (p = 0.048) was found in this group. In the Abx group, the absolute abundance of *Stenotrophomonas* (p = 0.000031), unknown *Enterobacteriaceae* (p = 0.0000514) and *Brevundimonas* (p = 0.036) was significantly increased, while the absolute abundance of *Streptococcus* (p = 6.21 × 10^−16^), *Lachnoclostridium* (p = 1.70 × 10^−15^), *Enerorhabdus* (p = 3.39 × 10^−14^), unknown *Lachnospiraceae* (p = 6.15 × 10^−13^), unknown *Ruminococcaceae* (p = 1.16 × 10^−11^), *Faecalibaculum* (p = 3.02 × 10^−11^), unknown Bacilli (p = 2.88 × 10^−7^), unknown *Muribaculaceae* (p = 0.0000212) and *Colidetribacter* (p = 0.00015) was significantly decreased. In the Abx + HFD group, the absolute abundance of *Stenotrophomonas* (p = 2.07 × 10^−6^), *Brevudimonas* (p = 0.02) and unknown *Enterobacteriaceae* (p = 0.04) was significantly higher than the ND group, while the absolute abundance of *Lachnoclostridium* (p = 2.57 × 10^−25^), *Enterohabdus* (p = 2.13 × 10^−19^), unknown *Lachnospiraceae* (p = 4.5 × 10^−17^), unknown *Rumonococcaceae* (p = 2.28 × 10^−16^), unknown *Muribaculaceae* (p = 1.47 × 10^−15^), *Lactobacillus* (p = 3.02 × 10^−15^), *Streptococcus* (p = 5.36 × 10^−12^), unknown Bacilli (p = 3.43 × 10^−7^), *Colidextribacter* (p = 3.54 × 10^−7^), *Parasutterella* (p = 2.04 × 10^−6^), *Enterococcus* (p = 0.000015) and *Akkermansia* (p = 0.00007) was significantly lower. HS intake significantly increased the absolute abundance of *Bifidobacterium* (p = 2.04 × 10^−20^), *Eubacterium* (p = 6.45 × 10^−11^), *Stenotrophomonas* (p = 0.0003), *Dubosiella* (p = 0.001), unknown Clostridia (p = 0.0013), *Nesterenkonia* (p=0.0018), unknown *Xanthomonadaceae* (p = 0.026), *Faecalibaculum* (p = 0.034) and *Halomonas* (p = 0.034) and significantly decreased *Streptococcus* (p = 6.45 × 10^−11^), unknown Bacilli (p = 1.13 × 10^−8^), *Colidextribacter* (p = 4.43 × 10^−6^), *Parasutterella* (p = 4.43 × 10^−6^), *Enterococcus* (p = 0.0003), *Pelagibacterium* (p = 0.003), and unknown *Enterobacteriaceae* (p = 0.02). In HS + HFD, the absolute abundance of *Bifidobacterium* (p = 4 × 10^−15^), *Eubacterium* (p = 4.4 × 10^−7^), *Anoxybacillus* (p = 0.00009), *Faecalibaculum* (p = 0.00017), and *Stenotrophomonas* (p = 0.023) was significantly higher than the ND group, while the absolute abundance of *Phyllobacterium* (p = 4 × 10^−15^), unknown *Muribaculaceae* (p = 6.68 × 10^−12^), *Streptococcus* (p = 4.77 × 10^−10^), unknown Bacilli (p = 2.14 × 10^−8^), *Colidextribacter* (p = 3.26 × 10^−6^), *Parasutterella* (p = 3.33 × 10^−6^), *Enterococcus* (p = 0.000042), *Akkermansia* (p = 0.0005), *Enterohabdus* (p = 0.002), unknown *Lachnospiraceae* (p = 0.02) and *Lactobacillus* (p = 0.02) was significantly lower.

**Table 2:** Numbers of significantly different taxa between treated group and ND group.

		Increase						Decrease					
		HFD		HFD + HS		HFD + Abx		HFD		HFD + HS		HFD + Abx	
		AA	RA	AA	RA	AA	RA	AA	RA	AA	RA	AA	RA
DADA2	Silva	12	1	5	0	18	0	6	0	12	2	13	5
	Greengenes	8	0	4	0	17	0	5	0	11	1	9	4
Deblur	Silva	9	0	2	0	8	1	7	0	11	2	12	6
	Greengenes	5	0	3	0	5	1	10	0	13	2	13	7
UNOISE3	Silva	3	1	5	0	3	1	2	0	11	6	13	9
	Greengenes	3	1	5	2	3	4	1	0	9	5	10	7
UPARSE	Silva	19	0	14	1	16	1	15	0	22	7	20	8
	Greengenes	13	1	12	1	13	1	12	0	15	4	14	9

(AA, calculation based on absolute abundance; RA, calculation based on relative abundance).

### Bacterial indicators for obesity and FBG level

We identified the significantly different jejunal bacteria between groups with significantly increased body weight (HFD) and others (ND, Abx, HS, Abx + HFD, and HS + HFD), and between groups with significantly elevated FBG levels (HFD, Abx + HFD, and HS + HFD) and with normal FBG levels (ND, Abx, and HS), and analyzed their correlation of absolute abundance with the relevant measures.

Along with the significant increase in BW, the absolute abundance of *Dubosiella* (p = 1.74 × 10^−10^), *Eubacterium* (p = 3.84 × 10^−10^), unknown *Enterobacteriaceae* (p = 3.84 × 10^−10^), *Parasutterlla* (p = 4.28 × 10^−10^), *Rombuotsia* (p = 1.65 × 10^−9^) and unknown Clostridia (p = 1.93 × 10^−8^) was significantly decreased, whereas the absolute abundance of *Colidextribacter* (p = 0.01) and *Faecalibaculum* (p = 0.04) was significantly increased. The absolute abundance of *Colidextribacter* (R = 0.695, p = 0.001) and *Faecalibaculum* (R = 0.631, p = 0.005) was significantly correlated with BW changes.

In groups with significantly elevated FBG levels, the absolute abundance of *Bifidobacterium* (p = 4.02^−28)^, *Dubosiella* (p = 6.46 × 10^−23^), *Eubacterium* (p = 6.46 × 10^−23^), *Faecallibaculum* (p = 1.24 × 10^−8^), unknown Clostridia (p = 0.00077), and *Colidextribacter* (p = 0.008) were significantly higher, while the absolute abundance of *Parasutterella* (p = 1.45 × 10^−22^), *Escherichia* (p = 0.0038), and unknown *Enterobaceriaceae* (p = 0.021) was significantly lower. Among these significant different bacteria, *Eubacterium* (R = 0.069), *Faecallibaculum* (R = 0.155), unknown Clostridia (R = 0.053), and *Colidextribacter* (R = 0.211) were correlated with FBG level but without significance of difference (p >0.05).

### Jejunal microbiota regulates T2DM *via* glucose degradation

We explored the changes in bacterial metabolism caused by HFD, antibiotics, and HS using PICRUSt, which predicts the changes in genes and KEGG pathways, and the significant changes in the pathway are shown in **Supplementary Figure 3**. Generally, the KEGG pathway of carbohydrate degradation is significantly affected by both diet and microbiota perturbation in the jejunum. Compared to a normal diet, the HS group, with the same nutrition formula, had the jejunal microbiota with a significantly downregulated predicted pathway of glucose and xylose degradation (p = 0.025), but the Abx group had no significant change (p >0.05). Similar to the formula of HFD, which contained high fructose, the HS + HFD group had a significantly upregulated pathway of glucose and glucose-1-phosphate degradation (p = 0.027) compared with the HFD group, whereas Abx + HFD had a significantly downregulated pathway of glucose and xylose degradation (p = 0.012). Although very small significant differences were found for the degradation of glycogen, antibiotics still enhanced this pathway compared to other groups. Besides, the pathway of carbohydrates in the jejunum was influenced by different treatments. Other pathways related to energy metabolism (e.g., NAD biosynthesis, TCA cycle), synthesis of structural compounds (e.g., histidine, purine, and pyrimidine biosynthesis), and nutrition biosynthesis (e.g., biotin), also changed significantly due to the shifts in jejunal microbiota.

## Discussion

In this study, we compared the shifts of microbiota between small and large intestines *via* perturbation using antibiotics and high salt intake, respectively. Under both perturbations, the growth trajectories of DIO mice and the development of T2DM were indeed altered. The jejunal microbiota was more dispersed within a group than the colonic microbiota. Moreover, based on jejunal microbiota profiled using four bioinformatics pipelines, we observed altered metabolic phenotypes accompanied by shifts in perturbed jejunal microbiota.

The presence of gut microbiota determines the development of HFD-induced obesity using AIMD and germ-free mice in previous studies (2, 26). Although similar results were observed in our study, some differences were noted concerning the ratio of the phylum. In this study, neither the Firmicutes to Proteobacteria (F/P) nor the Firmicutes to Bacteroidetes ratio (F/B) in the jejunum was significantly increased in HFD mice. This indicates that Firmicutes, dominating in the distal intestines of DIO mice and dietary obese patents (27, 28), are restrained in the proximal jejunum by antibiotics. Thus, the change in microorganisms in the jejunum is not the same as that in the distal intestine, including the ileum, cecal, colon, and even feces (28–30). Besides AIMD, our study introduced high salt intake, which is also one of the unhealthy eating habits associated with diet-induced obesity and T2DM (31, 32), as another perturbation for the gut microbiota. Both the metabolism and the gut microbiota of DIO mice were changed due to high salt intake, suggesting the HS intake is a suitable perturbation for studying the relationship between gut microbiota and metabolic disorders. On the one hand, high salt intake is alleged to cause metabolic disorders *via* endogenous fructose production in the aldose reductase–fructokinase pathway (32, 33). Our results agree with HS mice fed a normal diet who have elevated FBG levels. On the other hand, high salt intake reduces high-fat diet-induced obesity by changing the gut microbiota (31, 34). We also verified thisperspective since HS + HFD mice did not manifest obesity but had altered gut microbiota. Therefore, the perturbation of high salt intake added value to the illustration of the relationship between gut microbiota and T2DM without obesity manifestation.

Concerning T2DM, the mice in the HFD, HS, and HS + HFD groups manifested relevant symptoms. Fed with a normal diet, the HS group had hyperglycemia and unique jejunal signatures with significantly downregulated glucose and xylose degradation, suggesting more glucose remained for host absorption. The carbohydrate metabolism pathway of gut microbiota can also end with producing SCFAs, which supply additional energy sources for the host in the distal intestine (4). Meanwhile, gut microbiota in the small intestine regulate host metabolism *via* dietary lipid digestion and absorption (13). In this pilot study, we confirmed that the alteration of the carbohydrate metabolic pathway in jejunal microbiota is causally linked with the development of T2DM in DIO mice *via* several perturbation strategies. Together, we postulate that gut microbiota in the proximal small intestines regulate blood glucose *via* their glucose metabolic pathway. More details, including regulators of glucose metabolism, specific bacteria in glucose degradation, end-products of the altered pathway, and evaluation *via* microbiota transplantation, should be further explored.

Previous studies revealed that the relative abundance of *Akkermansia muciniphila* remarkably decreased in DIO mice and obese individuals, and supplements with this bacterium have the property of alleviating metabolic disorders (35, 36). The absolute abundance of *Akkermansia* also significantly decreased in the jejunal microbiota perturbed by the combination of HFD and HS or antibiotics in this study. Moreover, *Lactobacillus*, like other studies (31, 37), was reduced when these perturbations were applied. However, these reductions were observed only in mice with intensive perturbation, specifically a combination of HFD and antibiotics or HS intake, rather than when only HFD was applied. A previous study declared that gut microbiota present significant alteration after 24 weeks rather than 12 weeks in HFD-induced mice (38), and it might be a reason that minor changes in HFD fed mice without more intensive perturbation. Moreover, *Bifidobacterium*, of which the relative abundance decreased due to diet-induced obesity in other studies (5, 13), increased in the HFD and HFD + HS groups in our study concerning absolute abundance. In this study, other groups failed to detect *Bifidobacterium*, the same result with all pipelines, and this principally caused the significance of the difference. Technical limitations, such as PCR bias and bases mispairing, might be explanations for this controversial observation. Hypothetically, since *Bifidobacterium* has the property to repair the integrity of the intestines (39, 40), the significant increase in this genus in HFD and HS-fed mice might act as an early compensation to maintain the intestinal integrity.

Furthermore, we identified another two genera as indicator bacteria for obesity and T2DM using correlation analysis on the absolute abundance, namely, *Colidextribacter* and *Faecalibaculum* (identified as “*Allobaculum*” in the Greengenes database). Their absolute abundance was positively correlated with obesity and hyperglycemia. In a previous study, *Colidetribacter* could indicate the antioxidant capacity of the colon in mice (41). Although this genus was reported to be associated with obesity in previous studies, the further implication needs more extension. *Faecalibaculum* might become another promising marker genus. In a previous study, the abundance of *Faecalibaculum* decreased along with serum proinflammatory cytokines and lipopolysaccharide-binding protein (LBP) in HFD-fed mice, suggesting that it is involved in the gut proinflammatory pathway, which is associated with metabolic disorders (42). Our study established a link between the abundance of *Faecalibaculum* and obesity and T2DM, which agrees with the previous studies (42, 43); further application is still understudied.

Currently, next-generation sequencing is widely used to profile microbiomes in various fields, and studies revealing the relationship between gut microbiota and metabolic disorders have adopted 16S rRNA amplicon sequencing as a solid workflow. However, the outcomes of interpreting raw data were largely dependent on the bioinformatics analysis. In a previous study (15), UNOISE3 showed the best balance between resolution and specificity, as in ours. The ASV number generated from UNOISE3 was the lowest but covered the same taxa as others in our dataset. Moreover, the diversity analyses using UNOISE3 showed the utmost overlap with other pipelines; thus, we present our primary data analyzed using UNOISE3. Even so, there is much more helpful information from other pipelines concerning the study aims. For instance, DADA2 showed very high resolution as several sequences could not be identified, meaning more detailed taxonomic information might be dug out using these datasets. This same outcome was also observed in other studies, and DADA2 is used as a tool for exploring new taxa (44, 45). OTU-level is a traditional workflow widely used in commercial analyses; however, its low specificity and spurious OTUs, as well as inflated alpha-diversity measures, should be avoided (15). Therefore, we advise researchers to prudently choose a proper analysis workflow for 16S amplicon sequencing raw data to interpret the concept of their studies best.

In conclusion, the onset of obesity and T2DM in DIO mice was influenced when gut microbiota were perturbed. Moreover, glucose degradation might be the main pathway in which jejunal microbiota regulate the host metabolism, which must be substantiated further. Prospectively, the perturbation of gut microbiota can relatively ameliorate metabolic disorders, and it is promising to develop a new strategy for manipulating gut microbiota in clinical practice.

## Data availability statement

The datasets presented in this study can be found in online repositories. The names of the repository/repositories and accession number(s) can be found in NCBI PRJNA773977

## Ethics statement

This study was reviewed and approved by the Ethics Committee for Animal Research, Shenzhen Institutes of Advanced Technology, Chinese Academy of Sciences.

## Author contributions

ZY and P-GR designed the study. X-FY and GK performed the experiments. ZY analyzed the data. ZY, P-GR, and X-FY compiled the manuscript. All authors listed have made a substantial, direct, and intellectual contribution to the work and approved it for publication.

## Funding

This work was supported by the National Key Research and Development Program of China Grants (2021YFA0719303), the fellowship of China Postdoctoral Science Foundation (2021M703368), the Shenzhen Fundamental Research Program (KCXFZ20201221173400002), Shenzhen Science and Technology Program (JCYJ20200109115441918), Nation Natural Science Foundation of China (32100572).

## Conflict of interest

The authors declare that the research was conducted in the absence of any commercial or financial relationships that could be construed as a potential conflict of interest.

## Publisher’s note

All claims expressed in this article are solely those of the authors and do not necessarily represent those of their affiliated organizations, or those of the publisher, the editors and the reviewers. Any product that may be evaluated in this article, or claim that may be made by its manufacturer, is not guaranteed or endorsed by the publisher.
