# Supplementary material for: Perturbation on gut microbiota impedes the onset of obesity in high fat diet-induced mice
Source: Front Endocrinol (Lausanne). 2022 Aug 9;13:795371. doi: 10.3389/fendo.2022.795371 (PMC9395671; doi:10.3389/fendo.2022.795371)
Supplement: Supplementary file 1 [file DataSheet_1.docx]

Supplementary Material

**Supplementary table 1 information of samples**

| No. Sample | Con. DNA | Copies of gene(log 10 copies/μgDNA) | Total Reads | Passed reads | | | |
| --- | --- | --- | --- | --- | --- | --- | --- |
|  |  |  |  | DADA2 | Deblur | Unoise3 | Usearch-cluster |
| ND1 | 1,15 | 4,67 | 43539 | 21106 | 17935 | 26994 | 27379 |
| ND2 | 1,86 | 5,00 | 42202 | 19608 | 16267 | 23246 | 22678 |
| ND3 | 0,99 | 5,26 | 33143 | 12864 | 12129 | 17004 | 15554 |
| HFD1 | 0,67 | 5,52 | 33039 | 16722 | 13728 | 20334 | 19548 |
| HFD2 | 1,02 | 4,75 | 35314 | 15593 | 11541 | 15172 | 16015 |
| HFD3 | 1,09 | 5,66 | 35747 | 15711 | 13283 | 17305 | 16320 |
| Abx1 | 2,64 | 6,56 | 30939 | 10910 | 12736 | 19409 | 19431 |
| Abx2 | 1,88 | 5,21 | 33752 | 13380 | 13594 | 19361 | 19057 |
| Abx3 | 2,54 | 7,11 | 47245 | 19741 | 15434 | 23226 | 23097 |
| HS1 | 2,44 | 6,69 | 49770 | 24955 | 19623 | 27999 | 27591 |
| HS2 | 4,18 | 5,63 | 48320 | 21277 | 13020 | 18903 | 18724 |
| HS3 | 3,68 | 5,83 | 48003 | 22303 | 12738 | 18670 | 17987 |
| Abx+HFD1 | 1,655 | 4,88 | 41190 | 19839 | 15356 | 21750 | 22115 |
| Abx+HFD2 | 1,21 | 4,43 | 48058 | 23454 | 19054 | 27889 | 28055 |
| Abx+HFD3 | 2,72 | 4,60 | 45648 | 22826 | 18193 | 26853 | 27361 |
| HS+HFD1 | 3,68 | 5,65 | 34567 | 15349 | 13430 | 20094 | 20027 |
| HS+HFD2 | 2,94 | 5,15 | 48460 | 21269 | 12544 | 20092 | 20378 |
| HS+HFD3 | 3,52 | 4,88 | 49218 | 23024 | 7547 | 10757 | 11001 |
| Total | / | / | 748154 | 339931 | 258152 | 375058 | 372318 |

Supplementary Material should be uploaded separately on submission. Please include any supplementary data, figures and/or tables. All supplementary files are deposited to FigShare for permanent storage and receive a DOI.

Supplementary material is not typeset so please ensure that all information is clearly presented, the appropriate caption is included in the file and not in the manuscript, and that the style conforms to the rest of the article. To avoid discrepancies between the published article and the supplementary material,

**
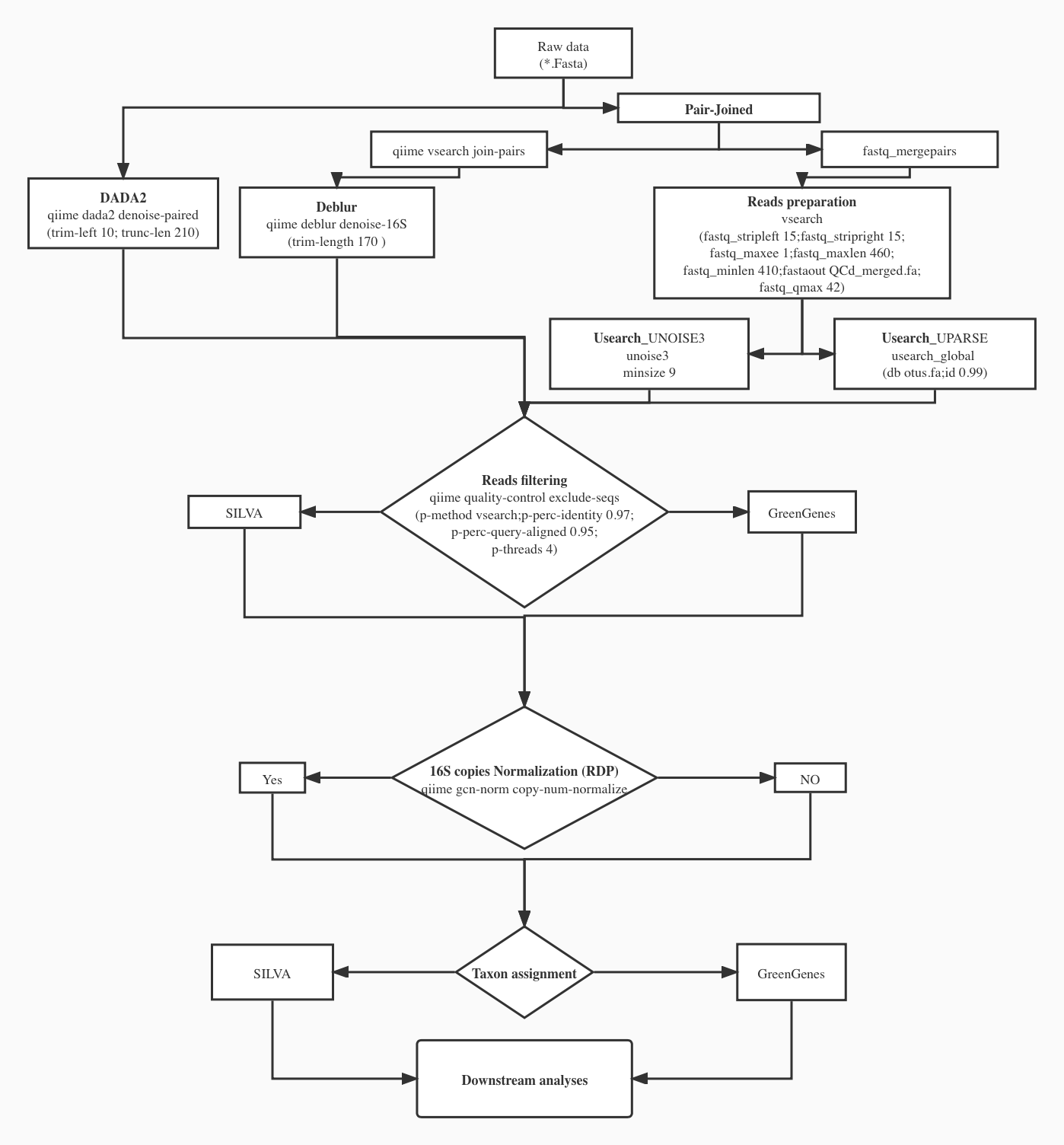
**

**Supplementary figure 1. Schema of bioinformatic analysis**

**Supplementary figure 2. Diversity index of jejunal microbiota identified using different parameters for bioinformatic analyses**. A. Alpha diversity; B. PCoA plot; C.Top 10 abundant taxon.

**Supplementary figure 3.** **Significantly different metabolic pathway of jejunal microbiota**.
